# Pathological diagnosis of ovarian cancer

**DOI:** 10.1186/1897-4287-10-S2-A82

**Published:** 2012-04-12

**Authors:** Hilda High, Michael Friedlander

**Affiliations:** 1 Hereditary Cancer Clinic, Prince of Wales Hospital, Randwick, NSW, 2031, Australia; 2 Medical Oncology, Prince of Wales Hospital, Randwick, NSW, 2031, Australia

## Case 1: Serous tubal cancer detected on PAP smear

A 60 year old woman with a history of breast cancer was referred after malignant cells were found on PAP smear. Abdominal exam, cervical visualisation, transvaginal ultrasound and CT of the abdomen and pelvis were all normal. Endocervical and endometrial curettings demonstrated serous carcinoma cells consistent with tubal / ovarian origin. CA125 was slightly elevated at 53 (Upper Limit of Normal 35).

At laparatomy uterus, fallopian tubes and ovaries appeared normal. She had a hysterectomy, bilateral salpingo-oophorectomy, omentectomy, peritoneal biopsies and washings. There was no macroscopic evidence of cancer, but microscopic examination confirmed serous cancer in the fimbrial end of the left fallopian tube(A) with scattered malignant cells in the lumen of both fallopian tubes(B), the omentum(C) and surface of the ovaries(D), surface of uterus and paracolic gutter. Washings were positive.

Family history was unremarkable. BRCA mutation screening revealed a BRCA1 mutation. Despite intraperitoneal cisplatin and paclitaxel she relapsed and died 4 years later.

## Case 2: Positive washing after RRSO in a BRCA 1 mutation carrier

A 40 year old woman had been diagnosed and treated for breast cancer at age 28. Mutation testing revealed a BRCA 1 mutation. She elected to have risk reducing salpingo-oophorectomy and hysterectomy at age 40. The fibria, fallopian tubes and ovaries were examined at 3mm intervals and immunohistochemistry was performed. The right fallopian tube was found to contain serous tubal intraepithelial carcinoma (STIC) with diffuse p53 staining (E) and Ki-67 seen in 50% of lesional nuclei (F). Washing were positive for adenocarcinoma and were similar in appearance to the serous tubal intraepithelial carcinoma. The left fallopian tube was found to have a precursor lesion with a p53 signature.

After discussion with her treating oncologist, and review of the case within the multidisciplinary tumour board, she elected to undergo adjuvant chemotherapy for ovarian cancer.

## Case 3: Right adnexal mass in residual right fallopian tube following bilateral oophorectomy

A 60 year woman presented with a right sided pelvic mass. In 2001 at age 48, she had been diagnosed with breast cancer. She underwent BRCA 1 or 2 Jewish founder mutation testing which revealed a BRCA1 mutation. She subsequently had risk reducing bilateral oophorectomy.

At laparatomy, the pelvic mass was a right adnexal mass which was a poorly differentiated serous carcinoma involving the right fallopian tube.

## Discussion

Knowledge of origin and evolution of ovarian cancer in BRCA mutation carriers has lead to changes in protocols of risk reducing surgery is performed and how specimens from risk reducing surgery are handled. A more intensive sectioning and staining as well as examination of peritoneal washing is now standard in most labs. These cases illustrate the tubal origin of BRCA-related serous cancers and the propensity for very early dissemination. In addition, pathology from previous gynaecological surgery should be reviewed to ensure complete removal of ovaries and fallopian tubes.

